# Implementing a new birthing room design: a qualitative study with a care provider perspective

**DOI:** 10.1186/s12913-023-10051-3

**Published:** 2023-10-19

**Authors:** Lisa Goldkuhl, Malin Tistad, Hanna Gyllensten, Marie Berg

**Affiliations:** 1 Institute of Health and Care Sciences, Sahlgrenska Academy, University of Gothenburg, Gothenburg, Sweden; 2 Department of Obstetrics and Gynaecology, Sahlgrenska University Hospital, Gothenburg, Region Västra Götaland Sweden; 3 School of Health and Welfare, Dalarna University, Falun, Sweden; 4 Department of Neurobiology, Care Sciences and Society, Karolinska Institute, Huddinge, Sweden; 5 Sahlgrenska Academy, University of Gothenburg Centre for Person-Centred Care (GPCC), University of Gothenburg, Gothenburg, Sweden; 6 Faculty of Medicine and Community Health, Evangelical University in Africa, Bukavu, Democratic Republic of Congo

**Keywords:** Complex interventions, Normalisation process theory, Implementation science, Qualitative research, Childbirth, Birthing room, Birth environment design, Care provider, Maternal health services

## Abstract

**Background:**

Research shows that interventions to protect the sensitive physiological process of birth by improving the birthing room design may positively affect perinatal outcomes. It is, however, crucial to understand the mechanisms and contextual elements that influence the outcomes of such complex interventions. Hence, we aimed to explore care providers’ experiences of the implementation of a new hospital birthing room designed to be more supportive of women’s birth physiology.

**Methods:**

This qualitative study reports on the implementation of the new birthing room, which was evaluated in the Room4Birth randomised controlled trial in Sweden. Individual interviews were undertaken with care providers, including assistant nurses, midwives, obstetricians, and managers (n = 21). A content analysis of interview data was conducted and mapped into the three domains of the Normalisation Process Theory coding manual: implementation context, mechanism, and outcome.

**Results:**

The implementation of the new room challenged the prevailing biomedical paradigm within the labour ward context and raised the care providers’ awareness about the complex interplay between birth physiology and the environment. This awareness had the potential to encourage care providers to be more emotionally present, rather than to focus on monitoring practices. The new room also evoked a sense of insecurity due to its unfamiliar design, which acted as a barrier to integrating the room as a well-functioning part of everyday care practice.

**Conclusion:**

Our findings highlight the disparity that existed between what care providers considered valuable for women during childbirth and their own requirements from the built environment based on their professional responsibilities. This identified disparity emphasises the importance of hospital birthing rooms (i) supporting women’s emotions and birth physiology and (ii) being standardised to meet care providers’ requirements for a functional work environment.

**Trial registration:**

ClinicalTrials.gov: NCT03948815, 14/05/2019.

**Supplementary Information:**

The online version contains supplementary material available at 10.1186/s12913-023-10051-3.

## Introduction

Both in Sweden and around the world, there is a trend towards an increase in the number of medical interventions during labour, such as caesarean births and the use of synthetic oxytocin for labour augmentation due to prolonged progress [1]. Overuse of medical interventions may lead to more risks than benefits [2], which is why the World Health Organization recommends preserving and supporting women’s physiological process of birth when providing intrapartum care [1]. Based on the knowledge that a perceived safe and comforting environment facilitates women’s complex hormonal childbirth processes [3], interventions to improve the built birth environment through features of familiarity and sensory stimulation have been initiated [4–8]. It is, for instance, known that a sense of unfamiliarity and stress inhibits the release of the neuropeptide oxytocin during labour, which can cause a stall in labour, increased pain sensations, and negative childbirth experiences [9, 10]. Studies have revealed that birthing rooms with calming lighting and multisensory elements, such as soothing images and sound effects, can reduce both caesarean birth rates [6] and the requirement for pharmacological pain relief [4, 8]. However, there are contradictory findings indicating no perinatal outcome effects when incorporating features to promote an upright birth position, as well as multisensory, and calming elements aimed at supporting birth physiology in hospital-based birthing rooms [5, 7, 11]. Nonetheless, a recent German randomised controlled trial (RCT) found that design interventions to promote mobility in the birthing room increased vaginal birth rates among both women in standard rooms and redesigned rooms, which may have been driven by increased motivation among women and care providers [7]. These findings highlight the need to provide a greater understanding about the mechanisms of impact of such complex research interventions [12].

Previous literature has described how the architectural design of healthcare facilities can impact the wellbeing, stress levels, healing processes and behaviours for all users, including admitted patients, companions, and care providers [13–15]. Factors such as noise reduction, favourable lighting and functional comfort in hospitals can also increase care providers’ quality of life at work [15, 16]. Since care providers can influence the healthcare environment by their presence, approach, support, and activities, they play a pivotal role in the quality of care [17, 18]. Therefore, it is essential that the birth environment design supports their ability to provide personalised care and addresses women’s emotional responses to the physiological process of birth. This is particularly important because social interactions and care practices also have major impacts on birth physiology [9]. For example, there is evidence showing that relation-based care, such as continuity-of-care models and continuous support during labour, can increase women’s chances of having a spontaneous vaginal birth and lead to a more positive childbirth experience. It also has a positive effect on pain perception and labour duration [19, 20].

This study reports on the implementation of a new birthing room, which was redesigned to support women’s physiological process of birth and was evaluated in the Swedish Room4Birth RCT [8]. Recruitment for the RCT started in 2019 but had an early discontinuation in 2020 due to the Covid-19 pandemic, resulting in only 406 of the 1274 planned study participants being included. The hypothesis that the new room would improve the primary composite outcome — spontaneous vaginal birth, no oxytocin augmentation, post-partum blood loss < 1000 ml and a positive childbirth experience in nulliparous women when compared to standard birthing rooms — could not be verified. Results from analyses of secondary outcomes showed that the new room contributed to women’s sense of safety, privacy and control, lowered the use of epidural analgesia, [8], and had a positive impact on childbirth experiences 3 and 12 months after birth [21].

The implementation of the new room was a complex intervention as it was embedded in a context with medico-technical requirements, human interactions, and individual and organisational needs. To understand how this complex intervention worked and to provide knowledge about the mechanisms that influenced its implementation [22], it is crucial to recognise how the users interacted with the birthing room. Labouring women’s use and experiences of the new room have been reported in previous Room4Birth studies [17, 21, 23]. The present study aimed to explore the care providers’ experiences of the implementation of the new birthing room.

## Methods

To gain an understanding of the implementation of the intervention evaluated in the Room4Birth RCT [8, 24], we adopted an explorative study design. Assistant nurses, midwives, obstetricians, and managers (hereafter referred to as ‘care providers’) who had experience of using the new room or were involved in its design development were interviewed individually. The study procedures and reporting of the manuscript followed the Consolidated Criteria for Reporting Qualitative Research (COREQ) checklist and a study protocol describing the full extent of the intervention has been published [24]. Moreover, the RCT conformed to the CONSORT guidelines and was registered at clinicaltrials.gov (14/05/2019, NCT03948815).

### Theoretical perspective

The study was informed by the Normalisation Process Theory (NPT), which seeks to understand how new practices become embedded or *normalised* within a context and the work that people do individually or collectively to enable the implementation of complex interventions [25, 26]. This theory was used to gain an understanding of the process of implementing the new room into everyday practice.

**Fig. 1:**
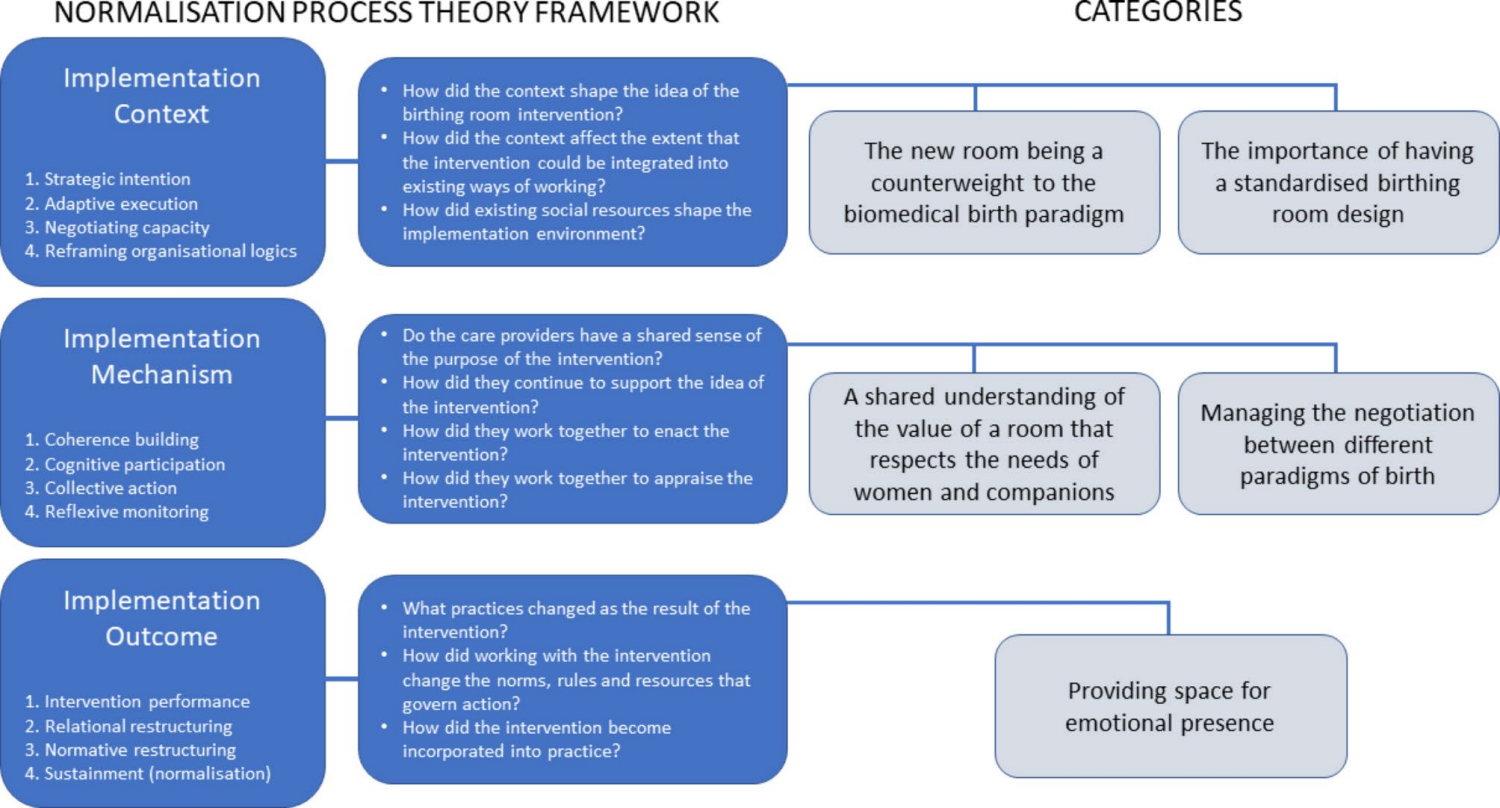
Map of categories organised from the 12 constructs of the Normalisation Process Theory coding manual [25]

In the original NPT framework, there are four primary constructs related to the mechanisms that influence the implementation and normalisation of interventions [26]. These constructs are: *Coherence building*, which reflects how the intervention makes sense to the participants; *Cognitive participation*, focusing on the work participants do to sustain practices around the intervention; *Collective action*, understanding how the intervention can be embedded into routine practice and work effectively; and *Reflexive monitoring*, which reflects the participants’ appraisal of the intervention. Recently, a coding manual for using the NPT framework in qualitative analysis has been developed [25]. This manual consists of 12 primary constructs (Fig. 1), which are linked to the realist evaluation framework [27] and are therefore structured around three domains: implementation context, mechanism, and outcome. This means that the coding manual takes into account the *mechanisms* described in the original NPT framework [26], but also how these mechanisms interact with considerations of an organisational *context* to produce intended *outcomes* [25].

### Setting

In Sweden, childbirth generally takes place in hospitals owned by the regions, in which the provided care is funded for all citizens through taxes. Birth without complications is independently handled under the responsibility of midwives. Obstetricians are consulted and are responsible in case of complication, but midwives remain the primary care provider throughout labour. Interprofessional teamwork between assistant nurses, midwives and obstetricians is key to the care provided during labour and these professions work in close collaboration [28]. The new birthing room was implemented at one of three labour wards at a Swedish University hospital. Approximately 10,300 babies are born annually at the hospital, with around 4,000 in the labour ward where the implementation occurred [29]. Apart from the new room, the labour ward had eight standard birthing rooms with a conventional design [24] and two infection isolating rooms accommodating birthing women with potential or confirmed infection. All rooms in the labour ward had private ensuite bathrooms with showers and toilets. The standard rooms resembled typical hospital rooms with centrally located birth beds and limited options for upright birth positions. Additionally, they lacked in-room bathtubs, had non-dimmable lighting, and featured medical equipment in full view.

### The birthing room intervention

The Room4Birth research project was closely linked to a project managing the planning and building of a new maternity clinic at the hospital. The new birthing room was implemented to study the effect of its design on perinatal outcomes, and to allow care providers to evaluate it prior to the start of the building process. The design process of the new birthing room was informed by previous research about healthcare environments [30, 31], and aspects raised by women who had given birth and their companions, by care providers representing all professions at the clinic, and by a layperson organisation.

The hypothesis for the RCT was based on the theory that a birthing room that meets personal needs reduces stress and facilitates the release of endogenous oxytocin [24]. This, in turn, enables labour to progress, reduces pain sensations, and stimulates innate nest-building behaviours [10]. The intervention included a physical redesign of the new birthing room, not a prespecified change in care providers’ practices. However, it was presumed that the new room could also impact their work environment and behaviours. With the aim of creating a soothing and more disarming environment, the new room was furnished with natural materials in earthy colours, soft corners on some surfaces, adjustable lighting, and medico-technical equipment concealed behind wooden panels. Sensory stimulation was offered through nature film projections on two walls, including music and nature sounds. Additionally, the design aimed to enhance mobility by featuring a secluded bed, providing in-room access to a bathtub, and offering alternatives for an upright birthing position, such as a Pilates ball, a trolley walker, and a birth support rope attached to the ceiling. Users of the new room had equal access to medical equipment, pharmacological analgesia, and medical interventions, including oxytocin augmentation and instrumental vaginal birth. Women in the new room were also provided care following the same guidelines as in the standard rooms. More details about the design and development process of the new birthing room have been published previously [24].

The care providers had the opportunity to familiarise themselves with the new room for one month prior to the start of the RCT. A study midwife, an assistant nurse, and four midwife study ambassadors covering day and night shifts were recruited to inform their colleagues about how to use the room and conduct the RCT study routines. This information was also provided by the first author of the paper (LG) who, as a doctoral candidate, was regularly present at the labour ward during the first year of the study.

### Data collection

A purposeful sampling of study participants was used and the number was determined to gain a variety and depth of understanding [32]. Inclusion criteria for care providers’ participation in the study were at least one year of working experience at the labour ward or having been involved in the design development of the new birthing room. Eligible participants were personally approached by the study midwife or LG during work shifts, via email or phone. Oral and written information about the study was given in accordance with ethical requirements. All midwives and assistant nurses who were informed about the study agreed to participate. Seven of the 11 obstetricians approached declined to participate since they considered that they did not have enough experience of using the new room. In total, 21 study participants were included, of which four (three midwives and one obstetrician) were working with hospital management and not in clinical practice (Table 1).

**Table 1:** Participant characteristics (n = 21)

Assistant nurses	5
Midwives	9
Obstetricians	3
Managers ^a^	4
Age, mean (range)	46 (33–62)
Professional experience in maternity care, years, mean (range)	14 (3–37)

^a^ Three midwives and one obstetrician

Individual audio-recorded, semi-structured interviews were carried out in Swedish five to eight months after the RCT was closed, by the study midwife (n = 19) or the first author (LG; n = 2). Both interviewers are midwives with experience of both childbirth care and conducting qualitative interviews. The interview guide included questions (Additional information 1) covering the original constructs of the NPT framework [26], and were found adequate after pilot testing. The interviews lasted from 30 to 67 min (mean: 46 min) and were conducted face-to-face (n = 3), by phone (n = 16), or video (n = 2) based on participant preference.

### Data analysis

The de-identified interviews were transcribed verbatim and listened to repeatedly to gain complete familiarity with the data. A two-stage approach was adopted for the analysis assisted by the NVivo software package (version 12). First, an inductive coding of the transcripts was carried out based on qualitative content analysis [33]. Meaning units of relevance for the research questions were identified, compared, and coded. Subsequently, the preliminary categories that emerged from the initial content analysis were mapped, interpreted, and rephrased in light of the recently published NPT coding manual and were thus structured around three domains: implementation context, mechanism and outcome [25]. This analysis method was used to allow codes to emerge from the data without being deductively determined by theory, and to organise and interpret the codes through the NPT framework. Coding and interpretation of data was led by LG, with frequent discussions and review of categories by all co-authors until full agreement. Quotations from the transcribed interviews are used to illustrate the research findings. These were translated from Swedish into English by the authors. The manuscript, including the quotations, were edited by a professional academic editing service.

## Results

Five categories, mapped into the NPT coding manual, describe the care providers’ experiences of the implementation of the new birthing room (Fig. 1).

### Implementation context

The findings within the implementation domain describe how the context affected the extent to which the intervention could be integrated into the existing environment. Categories included in the domain were — *The new room being a counterweight to the biomedical birth paradigm*, and *The importance of having a standardised birthing room design.*

#### The new room being a counterweight to the biomedical birth paradigm

The new birthing room was implemented at a labour ward where there was often a lack of rooms for birthing women, companions, and new-borns, and where care providers often experienced a heavy workload. The context consisted of collaborations between care providers with different professions and responsibilities representing different views on childbirth. It was acknowledged that the labour ward was dominated by a biomedical paradigm influenced by research and interventions to reduce medical risks in labour. This paradigm represented a view of birth as being an unpredictable and potentially critical process in need of technical and professional assistance. Some care providers emphasised the need for investment that incorporated subjective values respecting birth as primarily a healthy process. The new room materialised this health-promoting view and appeared as a counterweight to the prevailing biomedical paradigm focusing on pathology. The implementation of the new room reflected how the hospital organisation prioritised women and new families’ needs and wellbeing.

*Since we know that creating an environment that is perceived as safe and welcoming impacts the oxytocin levels in some sort of way (…) That’s what we aimed for with this room, that it would support natural birth, the non-medical. And it’s sort of a statement, that we focus on this and think this is important.* (P21, Manager)

#### The importance of having a standardised birthing room design

The care providers all found it problematic that the design of the new room differed markedly from the standard rooms. Since the labour ward had many employees and several birthing rooms, they did not use the new room often enough to become familiar with it. This unfamiliarity created a sense of insecurity, particularly in emergency situations where it was imperative not to waste time and energy feeling confused about where to find the needed equipment. The standard rooms all had a similar design, which was considered familiar and thus safer. The care providers reported that birthing rooms should be designed to support active birth and meet birthing women’s requirements but also cater to the needs of the care providers themselves. Therefore, standardisation of well-functioning birthing rooms was required where patient safety should never be compromised.

*You’re not used to the sounds and the films and all that* [in the new room]. *It feels a bit strange and unfamiliar to work in there. You need to think about where to find the things you need and adapt to the room. Not like all the other rooms where you just do your job.* (P3, Midwife)

Another aspect of having only one room in the labour ward with a unique design was that some of the care providers associated the new room with bad luck. They believed that one possible reason for this perception was that only nulliparous women used the new room during labour, as it was a recruitment criterion in the RCT. It was acknowledged that nulliparity was often associated with more complications than multiparity. Another potential explanation for why some care providers associated the new room with bad luck was that they specifically noted complications occurring in the new room, as it contradicted their idea of the health-promoting environment where women’s birth physiology ought to be supported.

*It’s a bit like, then there was a c-section, and you go “of course it was, because it’s in* [the new room]*”. It’s like there’s something going on, like something is haunted in there. That’s my feeling anyway. And maybe it’s because it’s only a single room that looks like that, and that’s why it just sticks in your memory. Because you would expect it to be the other way around.* (P13, Assistant nurse)

### Implementation mechanisms

The findings within the mechanism domain describe the care providers’ appraisal of the new room and the work that they did to integrate it as a part of ordinary practice. The implementation of the new room raised awareness about the importance of a more health-promoting view of birth, but it could also create a clash between the existing birth paradigms. Two categories were included in this domain — *A shared understanding of the value of a room that respects the needs of women and companions* and *Managing the negotiation between different paradigms of birth.*

#### A shared understanding of the value of a room that respects the needs of women and companions

The care providers all found the idea of the soothing elements in the new room — reminiscent of a home, a spa, or a hotel — beneficial for women during labour and birth. This enabled a sense of privacy and a healthy transition from the home to the hospital. Moreover, the flexibility of the room offered women and companions the ability to control their environment, thereby respecting subjective values. This was appreciated since the room could adapt to the needs of both those with a fear of hospitals, and those who expected and preferred an environment with fully visible medico-technical equipment. If women felt welcomed by a familiar environment, it was more likely that they would have trust in the professional decisions. The benefit of women choosing to give birth in hospitals was also emphasised. However, this was not seen as beneficial if the women were provided with unnecessary interventions, as described by an obstetrician.

*It might also promote childbirth in hospital, which I think is very positive. I think it’s safe and positive that people want to give birth in hospitals… but it’s not beneficial if they do it at the expense of us doing a lot of unnecessary things to them, or if we provoke situations that require interventions, which wouldn’t have been needed if it was in a different environment.* (P18, Obstetrician)

#### Managing the negotiation between different paradigms of birth

Having an alternative room at the labour ward reflecting a health-promoting view of birth had the potential to deepen the care providers’ understanding of the sensitive interplay between birth hormones and the surrounding environment. It opened their minds to ways and views of providing care other than those related to their own routine habits. The implementation of the new room could also prompt care providers to recognise that a standard room may be intimidating for people with limited experience in hospital contexts.

*I mean, you’re not aware of the existing structures until you have an alternative, really. You are shaped into a certain form already as a student, and as a midwife. It’s not even certain that you’re aware of how deeply ingrained these structures are until something challenges them* (P2, Midwife).

It was, however, recognised that the features within a room appreciated by women giving birth may not match those appreciated by care providers. For instance, the sounds and films deriving from the media installation in the new room provided positive distraction for women and companions, but it could be exhausting for the care providers to experience the same loop of films during a work shift. The analysis also identified that there were disparities in how the new room was perceived by different care providers, which seemed to be related to their professional responsibilities. For instance, some midwives emphasised the importance of having a secluded bed since it symbolised active birth, which was helpful in their efforts to support women’s birth physiology. On the other hand, it was stressed that childbirth required space for care providers to make their essential assessments and the secluded bed resulted in a lack of functionality. Some assistant nurses would describe the new room as occupied with too many unnecessary features, leading to a lack of space, and obstetricians described the idea of the new room as mainly the midwives’ domain, and not theirs. Since the room was impractical in emergency situations, even feelings of exclusion could be evoked. The health-promoting environment did not neatly align with obstetricians’ medical responsibility. For instance, it was challenging to disrupt the calm atmosphere of the new room by turning on the lights and to provide information about difficult medical decisions.

*I think there is a risk when things* [medico-technical equipment] *are hidden from us, when the rooms have been de-medicalised. And as soon as I enter the room, it all becomes very medicalised. We are used to all the instruments and all that. It is what we are working with. Then it becomes sort of a clash between our different environments. I think there is a slight risk to that as well.* (P17, Obstetrician)

### Implementation outcome

The outcome domain describes how the intervention changed care providers’ way of working and how this change was incorporated into daily practices. This domain included one category — *Providing space for emotional presence*.

#### Providing space for emotional presence

Some of the care providers addressed the need for greater organisational efforts to reverse the elevated rate of medical interventions, rather than only changing the birthing room design. The quality of care was mainly dependent on how the care was provided, and they were convinced that the new room did not change their practices. On the other hand, they described that the warm atmosphere of the room could make them slow down, absorb the nature scenes, and distance themselves from the hectic environment outside. Midwives felt that this could make them more physically present, but also more emotionally invested as it allowed them to follow the rhythm of labour and not only focus on check-ups and tasks. They acknowledged that their mental state of calmness could be transmitted to the birthing women.

*It was as if you stopped for a bit. You stayed in* [the new room] *longer because you were a little drawn into it, into the pictures. It was like this respectfulness, which I think we lack in the standard rooms. The hallway, the drapery, quite respectful. As if you enter the most sacred in some way (…) I think that if it’s an inviting atmosphere, with a lot of things to use and stress-relieving elements, where you can also allow yourself to follow the rhythm of birth, both me as a midwife and the parents benefit from that.* (P10, Midwife)

It was valuable that the new room also supported the companions to find their place in the room. For instance, the features promoting more upright birth positions made companions more active in providing support, which enabled the women to relax and cope with the labour pain. The new room also offered more non-pharmacological pain-relieving alternatives and the multisensory stimulation could be a way for care providers to initiate a conversation. Consequently, the new room was seen as a useful tool in the provision of care and relationship-building processes.

*I have more to offer, and in an easier way. I still try to make it comfortable in standard rooms as well and do it in a way that proves that you don’t have to be there* [in the new room]. *But there aren’t many other places to be, I don’t have that much to offer in standard rooms.* (P5, Midwife)

The view of birth embodied by the new room could also be transferred to the standard rooms. Care providers considered that design features that support women’s birth physiology should be provided for everyone and not only those allocated to the new room. Thus, the standard rooms were also gradually equipped with string lights, Pilates balls and trolley walkers after implementation of the new room.

## Discussion

The analysis of care providers’ experiences regarding the implementation of the new birthing room identified contextual elements, mechanisms and outcomes influencing the extent to which the room could be integrated as part of ordinary practice. Implementing the new room into the existing building raised the care providers’ awareness about the complex interplay between women’s birth physiology and the environment. This awareness had the potential to influence care providers to be more emotionally present with the woman and her companion, rather than to prioritise monitoring practices. However, the new room also evoked conflicting emotions as the care providers needed to negotiate between the health-promoting, personalised environment, and the prevailing biomedical paradigm of the labour ward context.

It is known that people conform to and interact with built environments, and it is through these human interactions that space is created [34, 35]. Redesigning environments has, therefore, been acknowledged as a strategy that can be used to support the implementation of new practices [36]. Altered care procedures were recognised as a potential consequence of the design change in the present study, even though the components of the intervention solely included a physical redesign. The interviewed midwives described how the new birthing room made them more physically present, and more emotionally invested. This illustrates how the new room could shift their focus from *doing* to *being*, a transition previously described as a move toward an embodiment of care that emphasises emotional presence over monitoring practices [37–39]. The concept of being ‘with woman’ and not ‘with the institution’ is considered fundamental in the practice and philosophy of midwifery. In addition to emotional presence, this concept encompasses building a partnership with the woman and her companion, thereby enhancing women’s agency [39]. Previous research has highlighted that care providers’ approaches and practices influence the atmosphere of the room, with considerable impacts on childbirth experiences and birth physiology [9, 17]. Being supported with emotional presence may have contributed to women randomised to the new room reporting more positive long-term childbirth experiences, as observed in the Room4Birth RCT [21], compared to women in standard rooms. Additionally, the midwives of the current study regarded the new room as a valuable resource in their endeavour to relieve women’s labour pain through non-pharmacological alternatives, which may partially explain the reduced use of epidural analgesia in the new birthing room [8]. Likewise, previous research has suggested that spatial planning can influence behaviours that indirectly impact the rate of medical interventions during labour [40] and midwives’ ability to support women’s physiological process of birth [41].

The health-promoting view of birth reflected in the new room corresponded with care providers’ perceptions of what is supportive for labouring women in their transfer from home to the hospital. As described in the NPT framework [25], participants need to understand the purpose of the intervention and construct value of its components for it to be successfully implemented. The fact that the care providers accepted the idea of the new design may have enabled them to use the room as intended. However, the unfamiliarity they experienced also gave rise to a sense of insecurity, which may explain why the new room was not fully integrated as a functional part of existing practice. Previous literature has described that insecurity within a setting can be challenging when providing intrapartum care, since managing the lack of familiarity takes time away from the woman [42], and leads to feelings of stress and discomfort [43]. It is crucial that the care providers can identify with their surroundings and that the room design matches their needs. Moreover, the design needs to be functional in emergency situations, yet prioritise women’s psychological wellbeing and physiological birth processes. These are aspects known to increase the effectiveness of and job satisfaction among care providers [16, 18, 43].

Our study demonstrates how the design of the new birthing room contrasted with the medico-technical environment in the standard rooms and the prevailing paradigm of the labour ward context. The new design aligned with a more integrative model of care, consistent with the social/midwifery model of care described in previous literature [9, 44]. This model understands birth from a health perspective and as being a process influenced by both neurobiological and psycho-social factors, in contrast to the biomedical model, which understands birth as being a mechanistic and potentially critical process in need of medical control [44]. Thus, the implementation of the new birthing room seemed to raise the care providers’ awareness about their usual understanding of birth, and about ways of providing care other than their routine habits.

Our findings also illustrate the challenge of implementing an intervention that was not entirely compatible with the established norms. Obstetricians and assistant nurses experienced a sense of exclusion or perceptions of the new room as occupied with irrelevant attributes, illustrating that the intervention was not fully aligned with their professional responsibility. These experiences contrasted with the midwives’ perception of the new room as helpful in their supportive activities and indicates conflicting preferences of what should be prioritised in built birth environments. Given that the new room reflected a more health-promoting view — which is in line with the scope of midwifery practice [45], rather than a biomedical view — this may have made the role of the obstetrician less clear. Our study did not illustrate that these contrasting views changed the power dynamics in the room. However, differing philosophical stances primarily between midwives and obstetricians have previously been shown to hinder interprofessional collaboration in maternity settings [46, 47]. This may also be a significant barrier to the implementation of the new room as an integrated part of daily practice [25], as all care providers regardless of profession and view, are essential team members of the care provided.

Some of the care providers in the current study associated the new room with complications, which seemed to be related to their unfamiliarity with the room. The main outcome of the Room4Birth RCT did not confirm these notions. The findings showed no increase in medical interventions or adverse events in the new room [8]. Instead, this perception may have been the result of cognitive bias and the product of the unblinded nature of the intervention. As new research interventions are introduced, people can have an increased awareness about related adverse events. This is also known as the Weber effect, originating from observations that adverse event reporting peaks after approval of new medicinal products, followed by a subsequent decline [48]. Once the room has been associated with complications, care providers may then interpret adverse events subjectively, or look for information to confirm their beliefs. Given that both the sense of insecurity and the association with complications seemed to stem from their unfamiliarity with the new room, the introduction of standardised birthing room designs, albeit with supportive features, should counteract this type of obstacle.

The design of the birthing room is one factor that may influence women’s experiences, birth physiology and care provision in a labour ward context. However, it is crucial to recognise that the prevailing paradigm and organisational decisions also play significant roles [18, 49, 50] — particularly since physical places carry cultural norms, which are conveyed to patients by care providers [35, 49]. Examples of this include surveillance technique behaviours and the use of medical interventions to maintain institutional efficiency at the expense of providing care tailored to personal needs and unique birth processes [51]. Designing birthing rooms with multisensory, ‘home-like’, and mobility-promoting elements has traditionally been a strategy in alternative birth settings, such as midwifery-led units. These settings prioritise the social/midwifery model of care over of the medical model, emphasising continuity of care and promoting personal control for women during childbirth [52]. In Sweden, hospital-based labour wards are the only integrated option within the healthcare system, and alternative models are rare. Given the significant role of the organisation of care, further development of both the birthing room intervention and the approach used to introduce it to care providers may have been necessary to facilitate reflection and a deeper understanding of how the birth environment influence women’s physiology and the care provided.

### Strengths and limitations

Using the NPT framework for the data collection and analysis was useful to understand care providers’ experiences of the implementation of the new room, as well as factors influencing the process of integrating it into daily practice [25, 26]. It also allowed an understanding of how the care providers appraised the room’s impact on their practices and, thereby, whether the intervention also influenced behaviours. A strength of using the updated NPT coding manual in data analysis is that contextual elements are taken into consideration, enabling the transferability of the results into similar contexts, although our results may not be transferable to labour wards with different organisational systems.

The individual interviews were conducted before the effectiveness of the intervention was known, which is a strength of the study. However, the results from the Room4Birth RCT were published by the time the analysis was undertaken, which could influence the interpretation of our findings. Trustworthiness of the study was achieved through repeated discussions involving all authors during the analysis process. It was also a strength that the authors represented different perspectives and that one of the authors (MT) was not involved in the effectiveness studies [8, 21]. Another strength was that the purposive sample of participants represented all professions at the labour ward, as well as the organisation of care. We purposefully chose to have most participants representing the midwifery profession since they spend the most time in the birthing room. However, it is a limitation that only four obstetricians participated, three of whom had experience of using the new room in clinical practice. The findings may, therefore, partially reflect a midwifery perspective. Moreover, it should be noted that the findings are based on care providers’ experiences, which may differ from how they actually utilised the new room, which requires an observation study.

## Conclusions

This study demonstrates that care providers found the birthing room intervention to be valuable and in alignment with their views of factors that support women’s birth physiology. The implementation of the new birthing room challenged the prevailing biomedical paradigm of the labour ward and had the potential to shift established care provider habits from routinised and task-focused behaviours towards integrating the practice and philosophy of being ‘with woman’. This change, aligned with the view of birth reflected in the new room, appeared to be facilitated by care providers’ recognition of the room’s value for women during childbirth. However, a barrier to the integration of the new room as a well-functioning part of ordinary practice was the feeling of insecurity evoked by its unfamiliar design. Another barrier was the perception that this design was not entirely functional in emergency situations and not fully compatible with medical responsibilities. It is evident that the birthing room design must be both aesthetically appealing and functional for care providers to find work satisfaction. We recommend that future design guidance for birth environments incorporate a combination of standardised, functional yet psychologically supportive room design that facilitates women’s birth physiology while promoting a sense of safety and familiarity among care providers.  Furthermore, future research should include interventions aimed at establishing practices that align with the sensitive interplay between the environment, personal experiences, and physiological birth processes.

### Electronic supplementary material

Below is the link to the electronic supplementary material.

Supplementary Material 1

Supplementary Material 2

## Data Availability

The data used and analysed during the current study are available from the corresponding author on reasonable request.

## Abbreviations

NPT: Normalisation Process Theory
RCT: Randomised Controlled Trial
