# Supplementary material for: Implementing a new birthing room design: a qualitative study with a care provider perspective
Source: BMC Health Serv Res. 2023 Oct 19;23:1122. doi: 10.1186/s12913-023-10051-3 (PMC10585888; doi:10.1186/s12913-023-10051-3)
Supplement: Supplementary file 1 — Supplementary Material 1 [file 12913_2023_10051_MOESM1_ESM.docx]

**Additional information 1**

**INTERVIEW GUIDE**

**IMPLEMENTING A NEW BIRTH ROOM DESIGN: A QUALITATIVE STUDY WITH A**

**CARE PROVIDER PERSPECTIVE**

**Background information**

What is your profession?

How old are you?

For how long have you been working in your profession in total?

The Room4Birth RCT started in January 2019. Have you worked at the labour ward since this time?

Do you have a specific responsibility at the labour ward?

Based on your own estimation, how many births have you attended in room 0?

**About the new room:**

- Tell me what it is like working in the new room compared with standard rooms at the labour ward. How does the room affect you? Describe whether you are doing anything differently.
- What value does this room have for you in your role as: midwife/assistant nurse/obstetrician/manager
- How relevant is this room for you in your professional role?
- What are the advantages or the disadvantages of the room design?
- What is the purpose and value of the room (the different physical features), according to your opinion?
- What is the value of the room for women and persons giving birth?
- What it the value of the room for the women´s companions?
- Were there consensus among the staff concerning:
- The functions of the room/the meaning of the features?
- The staffs´ experiences of working in the room?
- Tell me about your own – and your colleagues´ - commitment of using the room and the functions?
- How was the room integrated as part of daily practice?
- Describe if you were prepared for working in the room
- What was the best thing about working in the new room?

**Collaboration and relations in the labour ward:**

- Describe if the new room affected the collaboration among the care providers at the labour ward?
- Have new ways of collaboration between midwives, assistant nurses and doctors emerged?
- Has the new room affected the professional roles/power structures in any way?
- Describe if the new room affected the relationship among the care providers and the birthing women?
- Have the room affected the power structures between care providers and the women in any way?
- Describe if the new room affected the relationship among the care providers and the companions in any way?

**The effect of the new room:**

- Have you perceived any results or effects of the new room?
- Have you received any feedback about the effects of the room? Have you heard other people's stories? Describe.

**The room4birth study**

- What is your perception of the purpose of conducting the Room4Birth RCT?
- What is your perception of the meaning of the study?
- How relevant is this study for you in your professional role?
- Does this study have any value for you in your professional role? (as midwife/ assistant nurse/ obstetrician/ manager)
- What is the value of the study for birthing women?
- What is the value of the study for birth companions?

**Roles and responsibilities within the room4birth study:**

- Was there a common understanding of the value of the study for the staff? If not, describe
- Describe if there was a common understanding of how the study was introduced to the birthing women?
- Describe your own involvement in the study
- Describe your colleagues´ involvement in the study
- How did you perceive that the “key people” worked to run the study?

**Study routines:**

- How did the study routine work as part of daily practice?
- Were the study routines adapted to your way of working?
- Did you have knowledge and skills to work in the room and with the study procedures?
- Did you get opportunities to try the new room and the study procedures?
- Did you feel confident in your own abilities and safe in working in the room and with the study procedures?
- Did you have support from your managers?

**Development for the future:**

- How would you like the room to be designed to function more optimally? Do you have suggestions for improvement? Based on your experiences of working in the new room, what do you think could be improved in the room´s design?
